# Evaluating the Impact of Adverse Childhood Experiences on Brain Aging in Black and White individuals

**DOI:** 10.1002/alz70856_101095

**Published:** 2025-12-25

**Authors:** Abenaa Mensah‐Bonsu, Cristian O Mojica, Katelyn Elizabeth Mooney, Victoria N Poole, Melissa Lamar, Yingjuan Wu, Konstantinos Arfanakis, Lisa L. Barnes, Kacie D Deters

**Affiliations:** ^1^ University of California, Los Angeles, Los Angeles, CA, USA; ^2^ University of California, Los Angeles Neuroscience Interdepartmental Program (NSIDP), Los Angeles, CA, USA; ^3^ Rush Alzheimer's Disease Center, Chicago, IL, USA; ^4^ Rush Alzheimer's Disease Center, Rush University Medical Center, Chicago, IL, USA; ^5^ Rush University Medical Center, Chicago, IL, USA; ^6^ Illinois Institute of Technology, Chicago, IL, USA; ^7^ University of California, Los Angeles Integrative Biology and Physiology (IBP), Los Angeles, CA, USA

## Abstract

**Background:**

Adverse Childhood Experiences (ACEs) have been linked to a wide range of cognitive health issues. MRI‐based brain age prediction is a valuable tool for assessing brain health that has been made possible due to recent advancements in machine learning. These machine learning algorithms can predict an individual's brain age and how different it is from chronological age, a difference referred to as the Brain Age Gap (BAG). While research has explored various factors influencing brain aging, the impact of ACEs on BAG remains underexplored. Thus, this study aims to examine if childhood adversity is associated with accelerated brain aging in non‐Hispanic Black and White individuals.

**Method:**

Participants in this study consisted of cognitively normal older adults from the Rush Memory and Aging Project and the Minority Aging Research Study who self‐identified as Black (*N* = 233) or White (*N* = 619). Childhood adversity was categorized by the median split of low (Black = 0‐8; White = 0‐7) or high (Black = 9‐58; White = 8‐44). Linear regression analyses were then used to determine if childhood adversity was associated with BAG, including education and sex as covariates.

**Result:**

Higher levels of childhood adversity were significantly associated with higher BAG values (i.e., higher predicted brain age) in White individuals (*B* = 0.697, SE = 0.348, *p* =  0.046). No significant association was identified in Black individuals.

**Conclusion:**

This study provides preliminary evidence that childhood adversity may be linked to accelerated brain aging in White individuals. The lack of association in Black individuals may suggest other mechanisms, such as social or biological factors, that may influence brain aging in this group.

